# Radical enhancement of molecular thermoelectric efficiency†

**DOI:** 10.1039/c9na00649d

**Published:** 2020-01-26

**Authors:** Sara Sangtarash, Hatef Sadeghi

**Affiliations:** Physics Department, Lancaster University Lancaster LA1 4YB UK; School of Engineering, University of Warwick Coventry CV4 7AL UK hatef.sadeghi@warwick.ac.uk

## Abstract

There is a worldwide race to find materials with high thermoelectric efficiency to convert waste heat to useful energy in consumer electronics and server farms. Here, we propose a radically new method to enhance simultaneously the electrical conductance and thermopower and suppress heat transport through ultra-thin materials formed by single radical molecules. This leads to a significant enhancement of room temperature thermoelectric efficiency. The proposed strategy utilises the formation of transport resonances due to singly occupied spin orbitals in radical molecules. This enhances the electrical conductance by a couple of orders of magnitude in molecular junctions formed by nitroxide radicals compared to the non-radical counterpart. It also increases the Seebeck coefficient to high values of 200 μV K^−1^. Consequently, the power factor increases by more than two orders of magnitude. In addition, the asymmetry and destructive phonon interference that was induced by the stable organic radical side group significantly decreases the phonon thermal conductance. The enhanced power factor and suppressed thermal conductance in the nitroxide radical lead to the significant enhancement of room temperature *ZT* to values *ca.* 0.8. Our result confirms the great potential of stable organic radicals to form ultra-thin film thermoelectric materials with unprecedented thermoelectric efficiency.

## Abstract 2

The challenge in exploiting quantum interference (QI) in molecules for thermoelectricity lies in controlling the alignment of QI induced resonances close to electrodes Fermi level. We show that spin orbitals in stable radicals can be utilised to overcome this challenge.

## Graphical abstract

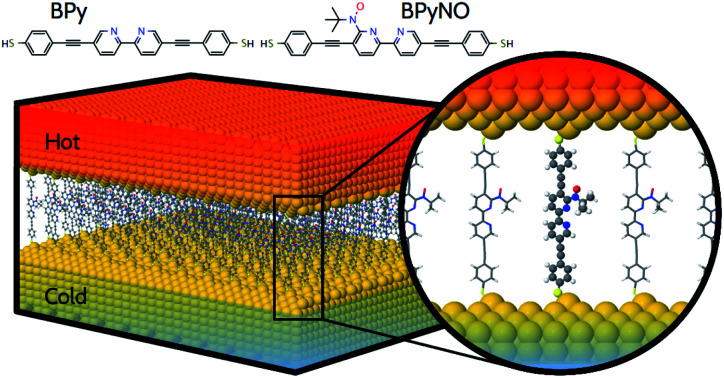

## Introduction

By 2030, twenty percent of the world's electricity will be used by computers and the internet, much of which is lost as waste heat.^1^ This waste heat could be recovered and used to generate electricity economically, provided materials with a high thermoelectric efficiency could be identified.^2–4^ Despite several decades of development, the state-of-the-art thermoelectric materials^5^ are not sufficiently efficient to deliver a viable technology platform for energy harvesting from consumer electronics or on-chip cooling of CMOS-based devices.^2,6^ The efficiency of a thermoelectric device is proportional to a dimensionless figure of merit^7,8^*ZT* = *S*^2^*GT*/*κ*, where *S* is the Seebeck coefficient, *G* is the electrical conductance, *T* is the temperature and *κ* = *κ*_el_ + *κ*_ph_ is the thermal conductance^9^ due to electrons *κ*_el_ and phonons *κ*_ph_. Therefore low-*κ*, high-*G* and high-*S* materials are needed. However, this is constrained by the interdependency of *G*, *S* and *κ*. Consequently, the world record *ZT* is about unity^5,10^ at room temperature in inorganic materials^11^ which are toxic (*e.g.* PbTe^12^) and their global supply is limited (*e.g.* Te).^13^ An alternative solution is to use organic molecular scale ultra-thin film materials.

In molecular scale junctions, electrons behave phase coherently and can mediate long-range phase-coherent tunneling even at room temperature.^14–17^ This creates the possibility of engineering quantum interference (QI) in these junctions for thermoelectricity. Sharp transport resonances are mediated by QI in molecular structures.^18^ This could lead to huge enhancements of *G* and *S* provided the energy levels of frontier orbitals are close to the Fermi energy (*E*_F_) of electrode. This is evident from high power factor (*S*^2^*G*) obtained by shifting *E*_F_ close to a molecular resonance in the C60 molecular junction using an electrostatic gating.^6,19^ However, using a third gate electrode is not desirable in a thermoelectric (TE) device because a TE device is expected to generate power but not to consume it through the electrostatic gating. An alternative solution would be to design molecular structures such that the energy level of frontier orbitals is pushed toward the Fermi energy (*E*_F_) of the electrode. In what follows, we demonstrate that this can be achieved using stable organic radicals.^20^ The single filled orbital in radicals has a tendency to gain or donate an electron and move down in energy; therefore, its energy level has to be close to the *E*_F_ of the electrode.

## Results and discussion

**Fig. 1:**
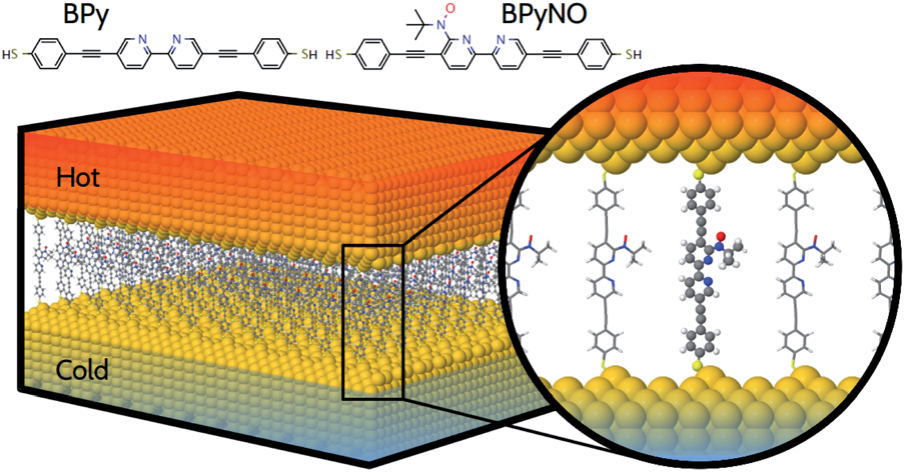
Molecular structure of a thermoelectric device where stable organic radical and non-radical molecules are placed between two hot and cold gold electrodes. Molecules consist of 2,2′-bipyridine (BPy) and 2,2′-bipyridine functionalized with *tert*-butyl nitroxide radical (BPyNO) cores connected to two thiobenzene anchors through acetylene linkers.

**Fig. 2:**
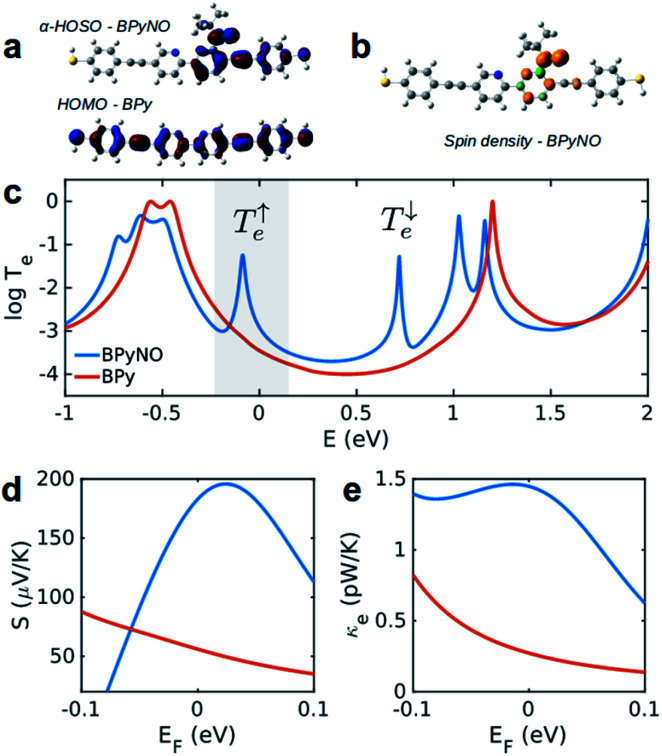
Electronic properties of BPy and BPyNO. (a) Highest occupied molecular orbitals of BPy and BPyNO. (b) Spin density in the BPyNO radical. (c) Transmission probability of electrons with energy *E* passing through BPy and BPyNO from one electrode to the other. (d) Seebeck coefficient and (e) thermal conductance due to electrons in BPy and BPyNO junctions *versus* the Fermi energy of the electrode at room temperature.

In this paper, we demonstrate that spin orbitals (SO) in nitroxide^21^ stable organic radicals can be used to design molecular structures with unprecedented thermoelectric efficiency. Fig. 1 shows the molecular structure of 2,2′-bipyridine (BPy) and 2,2′-bipyridine functionalized with *tert*-butyl nitroxide radical (BPyNO) cores connected to two thiobenzene anchors through acetylene linkers. BPyNO radicals have been demonstrated to be stable under ambient conditions with no decomposition for several months.^21^ In order to further enhance the stability of the molecular film formed by a massively parallel array of BPyNO, suitable encapsulation similar to that applied for 2D materials^22^ can be applied. BPy is a conjugated molecule and its highest occupied molecular orbital (HOMO) is extended over the molecule (Fig. 2a). The highest occupied spin orbital (HOSO) for majority spins of BPyNO is localized on the NO fragment and neighbouring phenyl ring (Fig. 2a). Spin density calculation (see Methods) reveals that this is due to the localization of majority spins on nitroxide radicals (Fig. 2b). Note that α-HOSO (highest occupied spin orbital), α-LUSO (lowest unoccupied spin orbital), β-HOSO and β-LUSO may be referred to also as spin-up HOMO, spin-up LUMO, spin-down HOMO and spin-down LUMO, respectively.

To study transport properties of junctions formed by BPy and BPyNO between the gold electrodes, we obtain material specific mean-field Hamiltonians from the optimised geometry of junctions using density functional theory (DFT).^23^ We then combine the obtained Hamiltonians with our transport code^7,24^ to calculate the transmission coefficient^7^*T*_e_(*E*) for electrons traversing from the hot electrode to the cold one (Fig. 1) through BPy and BPyNO (see Computational methods). *T*_e_(*E*) is combined with the Landauer formula^7^ to obtain the electrical conductance. At low temperatures, the conductance *G* = *G*_0_*T*_e_(*E*_F_) where *G*_0_ is the quantum conductance and *E*_F_ is the Fermi energy of the electrode. At room temperature, the electrical conductance is obtained by the thermal averaging of transmission coefficients calculated using the Fermi function (see Computational methods).

Fig. 2c shows the transmission coefficient *T*_e_(*E*) for electrons with energy *E* traversing through the BPy and BPyNO junctions. The red curve in Fig. 2c shows *T*_e_ for BPy. The room temperature electrical conductance of the BPy junction is *ca.* 4 × 10^−4^*G*_0_ at DFT Fermi energy (*E* = 0 eV). The electron transport is mainly through the HOMO level because of the extended HOMO state (see Table S1 of the ESI†). Furthermore, due to the charge transport between sulphur atoms and gold electrodes, in molecular junctions formed by thiol anchors, transport occurs to be through the HOMO state.^25^ Since the electronic structure of BPyNO is spin polarised, we compute the total 
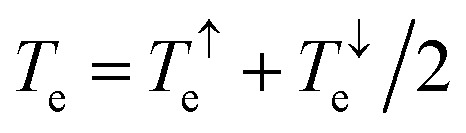
 from the transmission coefficient of majority (↑) and minority (↓) spins. The total *T*_e_ of the BPyNO junction is shown by the blue curve in Fig. 2c. Clearly, two new resonances are formed in the HOMO–LUMO gap of the parent BPy. These new resonances are due to the majority (↑) and minority (↓) spin orbitals localised on the nitroxide radical (see the orbitals of BPyNO and BPy in the ESI†). 
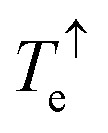
 and 
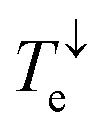
 for BPyNO radicals are shown in Fig. S1 of the ESI.†

**Fig. 3:**
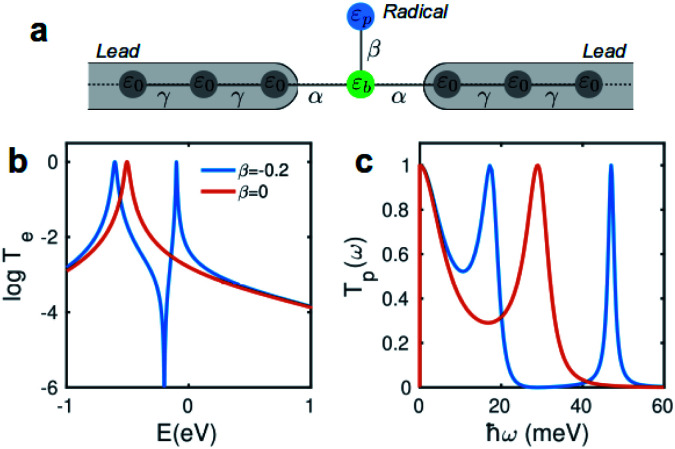
Tight-binding model. (a) A pendant group representing the singly occupied orbital is connected to a one level system. Transmission probability of electrons (b) and phonons (c) through the system with (*β* ≠ 0) and without (*β* = 0) pendant orbitals.

**Fig. 4:**
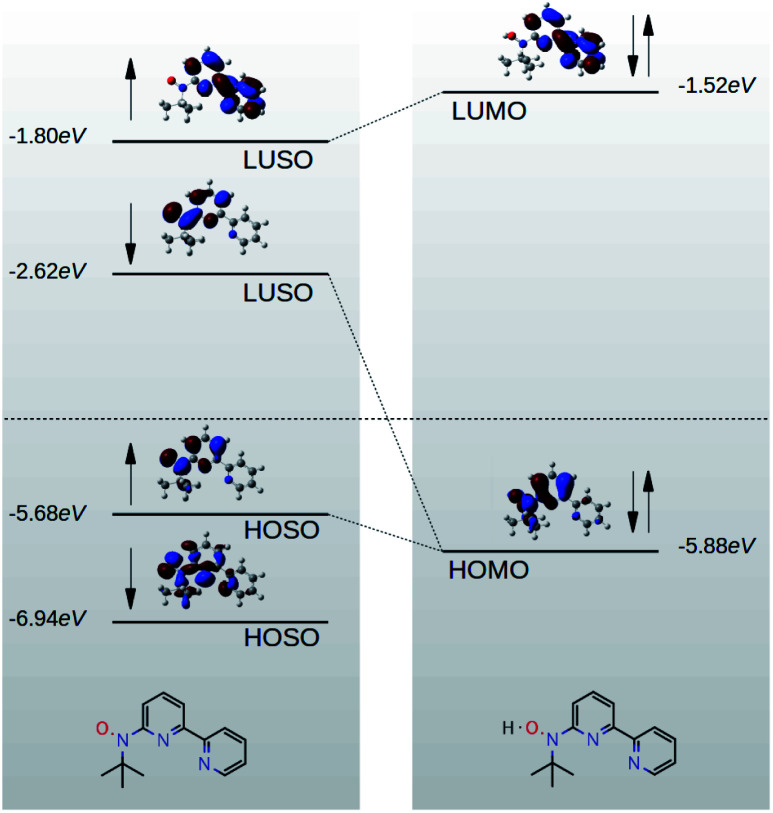
Orbitals of radical (left) and non-radical (right) BPyNO molecular cores.

Due to quantum interference between the transmitted wave through the backbone and reflected wave by the singly occupied orbital of the pendant group, a Fano-resonance forms. This is shown by the simple tight-binding model in Fig. 3b. When a pendant orbital is attached to the one level system (Fig. 3a), two resonances are formed due to the backbone and pendant sites. The resonances are close to the energy levels of these orbitals. The resonance due to α-HOSO is close to *E*_F_ in BPyNO (shown also with the grey region in Fig. 2c). The BPyNO radical has a tendency to gain (see Table S3 of the ESI†) an electron or share its electron (*e.g.* with a hydrogen atom to form –O–H) and minimize its energy. Fig. 4 shows the spin orbitals of the BPyNO molecular core and molecular orbitals of BPyNO with a hydrogen atom attached to oxygen to form the non-radical counterpart of BPyNO. When the hydrogen atom is detached from the core, the HOMO level of the non-radical BPyNO splits into two α-HOSO and β-LUSO states and moves up in energy.

The conductance of BPyNO is *ca.* 3 × 10^−3^*G*_0_ at DFT Fermi energy. Due to the new resonance transport through majority spins (see spin density plots in Fig. 2b), the conductance of BPyNO, on average, is about an order of magnitude higher than that of BPy around DFT Fermi energy. This is even higher closer to the resonance. This new resonance not only enhances the electrical conductance significantly, but also has a large effect on the room temperature Seebeck coefficient *S* (Fig. 2d). Note that *S* is proportional to the slope of the electron transmission coefficient *T*_e_ evaluated at the Fermi energy (*Sα*∂ ln *T*(*E*)/∂*E* at *E* = *E*_F_).^4,7^ As a consequence of the sharp slope of α-HOSO resonance in BPyNO close to *E*_F_, the Seebeck coefficient increases 4 times compared to that of BPy and reaches high values of *ca.* +200 μV K^−1^ in BPyNO. The sign of *S* is positive as a consequence of HOSO dominated transport in BPyNO.^26^

The heat is transmitted by both electrons and phonons.^3^Fig. 2e shows the thermal conductance due to electrons obtained from *T*_e_ in Fig. 2c (see Computational methods). The heat transport due to electrons is higher in BPyNO but its absolute value is very low in the range of 0.6–1.5 pW K^−1^ compared to other molecular junctions.^3,18^ In order to calculate thermal conductance due to phonons, we use material specific *ab initio* calculation. We calculate the transmission coefficient^7^ of phonons *T*_p_(*ω*) with energy *ℏω* traversing through BPy and BPyNO from one electrode to the other. The thermal conductance due to phonons (*κ*_p_) then can be calculated from *T*_p_(*ω*) using a Landauer like formula (see Computational methods).

**Fig. 5:**
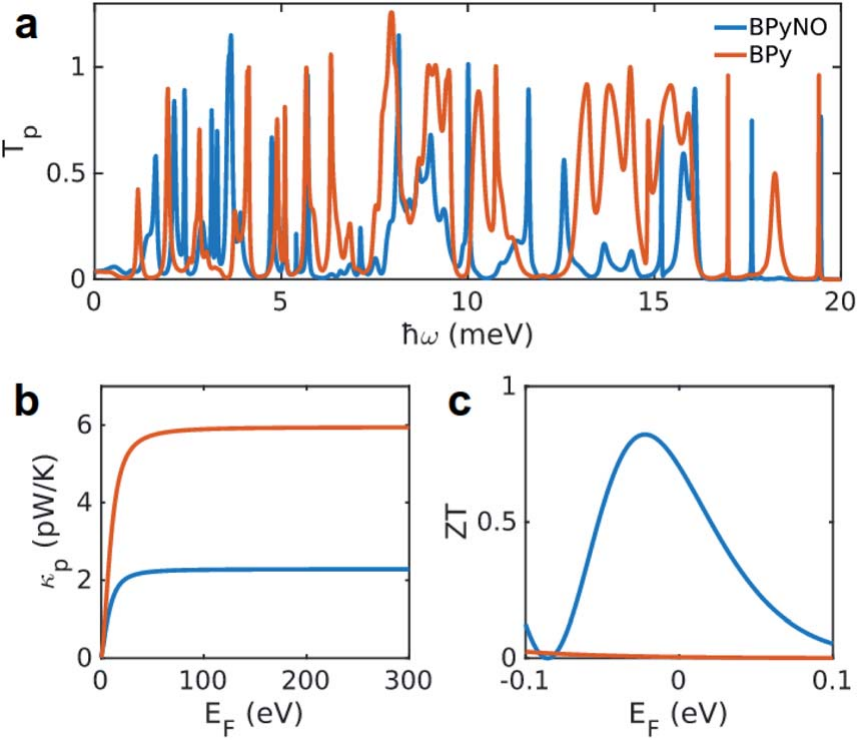
Phononic properties of BPy and BPyNO. (a) Transmission probability of phonons with energy *ℏω* passing through BPy and BPyNO from one electrode to the other. (b) Thermal conductance due to phonons in BPy and BPyNO junctions *versus* temperature. (c) Room temperature full *ZT* for BPy and BPyNO junctions *versus* the Fermi energy of the electrode.

Fig. 5a shows the phonon transmission coefficient *T*_p_(*ω*) for BPy and BPyNO junctions. Clearly *T*_p_ is suppressed in BPyNO compared to that of BPy for two reasons. First, the nitroxide radical makes the molecule asymmetric. Secondly, it reflects transmitting phonons through the BPy backbone. Consequently, the width of the resonances decreases.^7^ This is also confirmed by the simple tight binding model in Fig. 3c. Furthermore, some of the vibrational modes are suppressed *e.g.* modes at 6 meV, 9.5 meV and 13 meV (see movies in the ESI† that show the visualization of modes at these frequencies for both BPy and BPyNO). These two effects combined lead to a 3 times lower phonon thermal conductance in BPyNO (Fig. 5b). *T*_p_ is suppressed in BPyNO such that the electron and phonon contributions to the thermal conductance become comparable. We obtain the total room temperature thermal conductance of *ca.* 4.5 pW K^−1^ in BPyNO. The thermal conductance is dominated mainly by phonons in BPy leading to a total room temperature thermal conductance of *ca.* 6 pW K^−1^. From the obtained *G*, *S* and *κ*, we can now compute the full thermoelectric figure of merit^7^*ZT* as shown in Fig. 5c. *ZT* enhances significantly in the nitroxide radical functionalized junction (blue curve in Fig. 5c) compared to that of the parent BPy (red curve in Fig. 5c). A room temperature *ZT* of *ca.* 0.8 is accessible in the BPyNO radical for a wide energy range in the vicinity of *E*_F_. This is 160 times higher than room temperature *ZT* = 0.005 of BPy at *E*_F_.

Molecules are expected to show a high Seebeck coefficient because they pose sharp transport resonance features, thanks to their well separated discrete energy levels. However, a relatively small Seebeck coefficient has been measured in molecules so far.^3^ Among them, C60 shows the highest Seebeck coefficient of about −18 μV K^−1^ to −20 μV K^−4^. This leads to a power factor in the range of 0.03 pW per molecule. There is no thermal conductance measurement of C60 but using the predicted value,^27^ a low room-temperature *ZT* of 0.1 is expected. The challenge in exploiting quantum interference in molecules for thermoelectricity lies in controlling the alignment of the molecular levels and moving quantum interference induced resonances close to the Fermi level of the electrodes. Resonance transport close to the Fermi level through spin orbitals that we propose is a generic feature of stable organic radicals which can be utilised to overcome this challenge and enhance the thermoelectric efficiency of molecular junctions. The massively parallel array of BPyNO in self-assembled monolayers can then be formed to create ultra-thin molecular films with high *ZT* to convert waste heat to electricity.

## Conclusions

In this paper, we demonstrated for the first time that the thermoelectric figure of merit of junctions formed by the nitroxide stable radical enhances significantly from *ca.* 0.005 in the parent BPy to 0.8 in the daughter BPyNO. This enhancement is a generic feature of radicals because they create resonances close to the Fermi energy of the electrode. This ground breaking strategy can be utilized to design molecular junctions and ultra-thin film thermoelectric materials for efficient conversion of waste heat to electricity or on-chip cooling of CMOS-based technology in consumer electronic devices.

## Computational methods

### Geometry optimization

The geometry of each structure studied in this paper was relaxed to a force tolerance of 10 meV Å^−1^ using the *SIESTA*^23^ implementation of density functional theory (DFT), with a double-*ζ* polarized basis set (DZP) and the Generalized Gradient Approximation (GGA) functional with Perdew–Burke–Ernzerhof (PBE) parameterization. A real-space grid was defined with an equivalent energy cut-off of 250 Ry. To calculate molecular orbitals and spin density of gas phase molecules, we employed an experimentally parameterised B3LYP functional using Gaussian g09v2 (ref. 28) with a 6-311++g basis set and tight convergence criteria.

### Electron transport

To calculate the electronic properties of the junctions, from the converged DFT calculation, the underlying mean-field Hamiltonian *H* was combined with our quantum transport code, *Gollum*.^24^ This yields the transmission coefficient *T*_e_(*E*) for electrons of energy *E* (passing from the source to the drain) *via* the relationship *T*_e_(*E*) = Tr(*Γ*^e^_L_(*E*)*G*^R^_e_(*E*)*Γ*^e^_R_(*E*)*G*^R†^_e_(*E*)) where *Γ*^e^_L,R_(*E*) = i(*Σ*^e^_L,R_(*E*) − *Σ*^e†^_L,R_(*E*)) describes the level broadening due to the coupling between left L and right R electrodes and the central scattering region, *Σ*^e^_L,R_(*E*) is the retarded self-energy associated with this coupling and *G*^R^_e_ = (*ES* − *H* − *Σ*^e^_L_ − *Σ*^e^_R_)^−1^ is the retarded Green's function, where *H* is the Hamiltonian and *S* is the overlap matrix obtained from the *SIESTA* implementation of DFT. The DFT+Σ approach has been employed for spectral adjustment.^7^

### Phonon transport

Following the method described in ref. 7 and 8 a set of *xyz* coordinates were generated by displacing each atom from the relaxed *xyz* geometry in the positive and negative *x*, *y* and *z* directions with δ*q*′ = 0.01 Å. The forces *F*_*i*_^*q*^ = (*F*_*i*_^*x*^, *F*_*i*_^*y*^, *F*_*i*_^*z*^) in three directions *q*_*i*_ = (*x*_*i*_, *y*_*i*_, *z*_*i*_) on each atom were then calculated and used to construct the dynamical matrix 
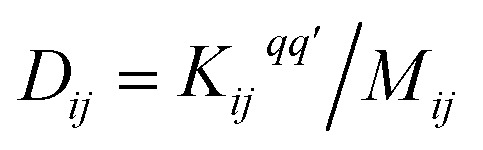
 where the mass matrix 
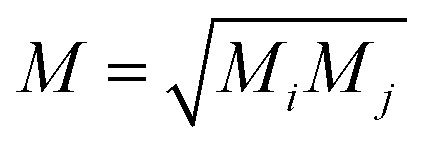
 and 
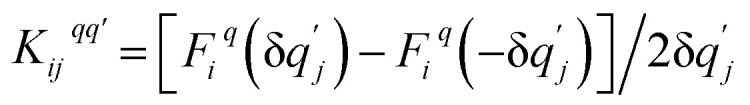
 for *i* ≠ *j* were obtained from finite differences. To satisfy momentum conservation, the *K* for *i* = *j* (diagonal terms) is calculated from 
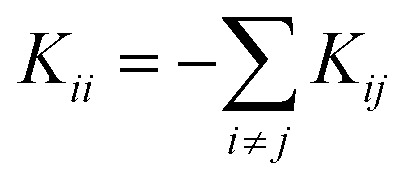
. The phonon transmission *T*_p_(*ω*) then can be calculated from the relationship *T*_p_(*ω*) = Trace(*Γ*^p^_L_(*ω*)*G*^R^_p_(*ω*)*Γ*^p^_R_(*ω*)*G*^R†^_p_(*ω*)) where *Γ*^p^_L,R_(*ω*) = i(*Σ*^p^_L,R_(*ω*) − *Σ*^p†^_L,R_(*ω*)) describes the level broadening due to the coupling to the left L and right R electrodes, *Σ*^p^_L,R_(*ω*) is the retarded self-frequency associated with this coupling and *G*^R^_p_ = (*ω*^2^*I* − *D* − *Σ*^p^_L_ − *Σ*^p^_R_)^−1^ is the retarded Green's function, where *D* and *I* are the dynamical and the unit matrices, respectively. The phonon thermal conductance *κ*_p_ at temperature *T* is then calculated from 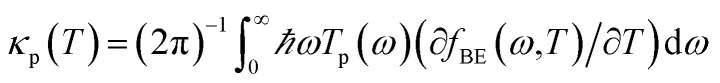 where *f*_BE_(*ω*,*T*) = (e^*ℏω*/*k*_B_*T*^ − 1)^−1^ is the Bose–Einstein distribution function and *ℏ* is reduced Planck's constant and *k*_B_ is Boltzmann's constant.

### Thermoelectric properties

Using the approach explained in ref. 7, the electrical conductance *G* = *G*_0_*L*_0_, the electronic contribution of the thermal conductance *κ*_el_ = (*L*_0_*L*_2_ − *L*_1_^2^)/*hTL*_0_ and the Seebeck coefficient *S* = −*L*_1_/*eTL*_0_ are calculated from the electron transmission coefficient *T*_e_(*E*) where the momentums 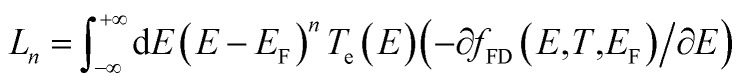 and *f*_FD_ is the Fermi–Dirac probability distribution function *f*_FD_ = (e^(*E*−*E*_F_)/*k*_B_*T*^ + 1) − 1, *T* is the temperature, *E*_F_ is the Fermi energy, *G*_0_ = 2*e*^2^/*h* is the conductance quantum, *e* is the electron charge and *h* is Planck's constant. The full thermoelectric figure of merit *ZT* is then calculated using *ZT*(*E*_F_,*T*) = *G*(*E*_F_,*T*)*S*(*E*_F_,*T*)^2^*T*/*κ*(*E*_F_,*T*) where *G*(*E*_F_,*T*) is the electrical conductance, *S*(*E*_F_,*T*) is the Seebeck coefficient, and *κ*(*E*_F_,*T*) = *κ*_el_(*E*_F_,*T*) + *κ*_ph_(*T*) is the thermal conductance due to the electrons and phonons.

## Data availability

The input files to reproduce simulation data can be found at https://warwick.ac.uk/nanolab.

## Conflicts of interest

There are no conflicts to declare.

## Supplementary Material

NA-002-C9NA00649D-s001

NA-002-C9NA00649D-s002

NA-002-C9NA00649D-s003

NA-002-C9NA00649D-s004

NA-002-C9NA00649D-s005

NA-002-C9NA00649D-s006

NA-002-C9NA00649D-s007
