# Supplementary material for: Radical enhancement of molecular thermoelectric efficiency
Source: Nanoscale Adv. 2020 Jan 26;2(3):1031–5. doi: 10.1039/c9na00649d (PMC9418312; doi:10.1039/c9na00649d)
Supplement: NA-002-C9NA00649D-s007 [file NA-002-C9NA00649D-s007.pdf]

Electronic Supplementary Material (ESI) for Nanoscale Advances.
This journal is © The Royal Society of Chemistry 2020
Please do not adjust margins

Nanoscale
Advances

ARTICLE

Supporting Information

Radical Enhancement of Molecular Thermoelectric Efficiency

*Sara Sangtarash[1] and Hatef Sadeghi[2,]**

[1] Physics Department, Lancaster University, Lancaster LA1 4YB, United Kingdom
[2] School of Engineering, University of Warwick, Coventry CV4 7AL, United Kingdom
**hatef.sadeghi@warwick.ac.uk*

Table S1. Spin orbitals of BPyNP radical

Level	spin	Energy (eV)	Spin orbitals of BPyNP radical
LUSO+2	α/↑	-1.28	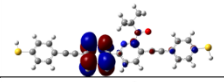
LUSO+2	β/↓	-1.54	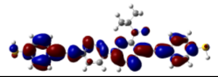
LUSO+1	α/↑	-1.57	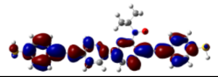
LUSO+1	β/↓	-2.37	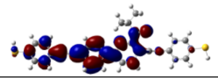
LUSO	α/↑	-2.48	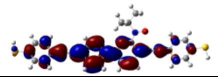
LUSO	β/↓	-2.73	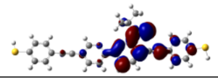
HOSO	α/↑	-5.44	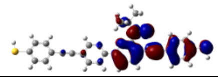
HOSO	β/↓	-5.92	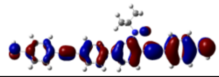
HOSO-1	α/↑	-6.17	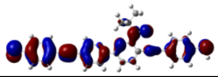
HOSO-1	β/↓	-6.26	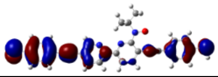
HOSO-2	α/↑	-6.29	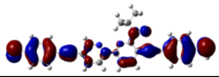
HOSO-2	β/↓	-6.67	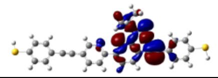

Please do not adjust margins

Table S2. Molecular orbitals of BPy

Level	Spin	Energy (eV)	Molecular orbitals of BPy
LUMO+2	↑↓	-1.17	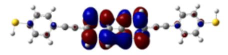
LUMO+1	↑↓	-1.63	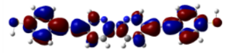
LUMO	↑↓	-2.36	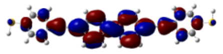
HOMO	↑↓	-5.98	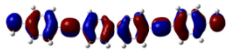
HOMO-1	↑↓	-6.26	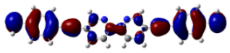
HOMO-2	↑↓	-6.91	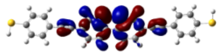

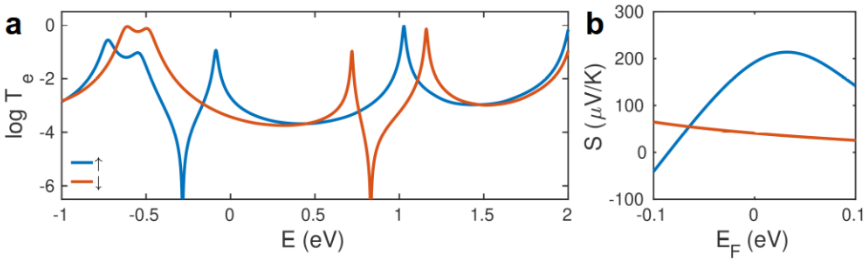

Fig. S1. (a) Transmission probability of electrons with different spin and energy *E* passing through BPyNO radical from one electrode to the other. (b) Room temperature Seebeck coefficient for majority and minority spins traversing through BPyNO radical versus electrodes Fermi energy.

Table S3. Total energy of BPyNO core in the different charge state. Adding an electron to this core is energetically easier than removing an electron from it.

Charge	0	-1	+1
Total Energy (eV)	-21293.38	-21294.64	-21286.14
Total Energy Differences (eV)	0	-1.26	7.24
